# Temperature sensitivity differs between heart and red muscle mitochondria in mahi-mahi (*Coryphaena hippurus*)

**DOI:** 10.1038/s41598-020-71741-0

**Published:** 2020-09-10

**Authors:** Gigi Y. Lau, Georgina K. Cox, John D. Stieglitz, Daniel D. Benetti, Martin Grosell

**Affiliations:** 1 Department of Zoology, University of British Columbia, 6270 University Blvd, Vancouver, BC V6T 1Z4 Canada; 2 Department of Marine Biology and Ecology, Rosenstiel School of Marine and Atmospheric Science, University of Miami, 4600 Rickenbacker Causeway, Miami, FL 33149 USA; 3 Department of Marine Ecosystems and Society, Rosenstiel School of Marine and Atmospheric Science, University of Miami, 4600 Rickenbacker Causeway, Miami, FL 33149 USA

**Keywords:** Animal physiology, Mitochondria

## Abstract

Maintaining energy balance over a wide range of temperatures is critical for an active pelagic fish species such as the mahi-mahi (*Coryphaena hippurus*), which can experience rapid changes in temperature during vertical migrations. Due to the profound effect of temperature on mitochondrial function, this study was designed to investigate the effects of temperature on mitochondrial respiration in permeabilized heart and red skeletal muscle (RM) fibres isolated from mahi-mahi. As RM is thought to be more anatomically isolated from rapid ambient temperature changes compared to the myocardium, it was hypothesized that heart mitochondria would be more tolerant of temperature changes through a greater ability to match respiratory capacity to an increase in temperature and to maintain coupling, when compared to RM mitochondria. Results show that heart fibres were more temperature sensitive and increased respiration rate with temperature increases to a greater degree than RM. Respiratory coupling ratios at the three assay temperatures (20, 26, and 30 °C), revealed that heart mitochondria were less coupled at a lower temperature (26 °C) compared to RM mitochondria (30 °C). In response to an in vitro acute temperature challenge, both tissues showed irreversible effects, where both heart and RM increased uncoupling whether the assay temperature was acutely changed from 20 to 30 °C or 30 to 20 °C. The findings from this study indicate that mahi-mahi heart mitochondria were more temperature sensitive compared to those from RM.

## Introduction

Mitochondria are the nexus of aerobic energy production within cells and maintain oxidative phosphorylation efficiencies to support metabolism over a wide range of physiological conditions. Temperature changes can impact mitochondrial function^1,2^ and an organism’s ability to maintain energy balance. Acclimation or adaptation to temperature changes, can lead to changes in mitochondrial function^3^ and efficiency^4,5^. Many pelagic species perform substantial daily vertical migrations as a foraging strategy^6–11^. As a result of this behaviour these species can experience rapid changes in temperature over short time scales (e.g. minutes) as they pass through the thermocline from warm surface waters into cooler deeper waters. While the diel patterns of depth distribution differs between species, this behaviour results in fish experiencing a wide range of temperatures regularly^7,9^. For example, mahi mahi (*Coryphaena hippurus*, from here on referred to as “mahi”) have been shown to make daily vertical movements of up to 111 m depth, and experience temperature changes of up to > 8 °C^6,7^. On a v-type dive where the fish abruptly descends then ascends in the water column, temperature changes can occur acutely with rates of up to 1 °C/min depending on the profile of the thermocline^7,9,11^. Our research group has recently collected data that confirms that tagged mahi experience this rate of temperature change naturally (unpublished data). Yet, how these drastic changes in temperature acutely affect mitochondrial function in this highly active species remain unknown.

Mahi are an active pelagic fish species that feed frequently to support high growth rates and energetic demands, exemplified by their high maximal metabolic rates^12,13^. Both cardiac and red muscle (RM) tissues are functionally important for mahi to maintain this active lifestyle. The sensitivity of these tissues to temperature changes that could be experienced by foraging mahi is particularly interesting given that previous studies on fish reported that the temperature at which heart failure occurs due to thermal stress coincides with the temperature at which mitochondria uncouple^14,15^. This loss of coupled mitochondria leads to decreased phosphorylation efficiency and the accumulation of reactive oxygen species that can cause oxidative damage in tissues^14,15^. In comparison to RM, the heart is likely to experience more rapid temperature changes as a result of coronary artery morphology. The coronary arteries branch from the efferent gill arteries, and therefore carry oxygenated blood directly from the gill to the heart. The coronary artery in this species perfuses ~ 50% of the cardiac tissue (Cox, GK, personal observation) and as blood exiting the gill is typically at a thermal equilibrium with the ambient environmental temperature, the heart itself could experience more rapid temperature changes than the more insulated RM.

This study was designed to test the effects of temperature on mitochondrial respiration in permeabilized heart and RM fibres from mahi. It was hypothesized that heart and RM mitochondria would be able to maintain function across a 10 °C range (20–30 °C), similar to what mahi experience naturally^6,7,10^. Further, it was predicted that heart mitochondrial function would be more tolerant and not uncouple in response to acute temperature changes compared to RM mitochondria.

## Results and discussion

Maintaining both cardiovascular and skeletal muscle function over a wide temperature range is extremely important for an active species that perform daily vertical migrations. On these dives, mahi rely heavily on aerobic ATP supply, which is highly dependent on the ability to maintain mitochondrial function^7,13^. As the thermal range that mahi can experience on a typical vertical dive is 10 °C (20–30 °C; unpublished data from satellite tags deployed by our group), it was thus predicted that as temperature increase from 20 to 30 °C mitochondria would be able to increase respiratory capacity and maintain coupling to meet increased energetic demands. Furthermore, it was predicted that heart mitochondria would be able to respond more rapidly to changes in environmental temperature and energetic demands when compared to RM mitochondria. Results from this study show that mahi heart mitochondria were indeed more temperature sensitive when compared to RM mitochondria, but lost phosphorylation efficiency as temperature increased.

### Permeabilized heart fibres were more temperature sensitive than RM fibres

Respiration rates of heart and RM fibres at the three assay temperatures (20, 26, and 30 °C) were expressed relative to ETS capacity to account for the variation of ETS capacity between fibres (see Supplementary Fig. S1 for respiration rate expressed to tissue weight; Fig. 2). In heart fibres, there were no effects of temperature on heart complex I-stimulated ADP-stimulated state 3 respiration rate (p = 0.06) or complex I and II-stimulated state 3 respiration rates (p = 0.14; Fig. 2A,B), but there was an effect of assay temperature on state 4_o_ leak respiration rate (p = 0.0005). In the RM fibres, there was no effect of assay temperature on complex I state 3 (p = 0.64), complex I and II stimulated state 3 (p = 0.94), or RM 4_o_ respiration rates (p = 0.83; Fig. 2). Heart leak respiration is significantly increased at 26 °C and so a substantial proportion of State 3 respiration rate (Fig. 2A,B) is able to compensate for this increase in proton leak. In contrast, RM leak respiration was unaffected by assay temperature and was more stable. Further, the heart Q_10_ values were generally higher compared to those from RM, which is consistent with heart mitochondria being more temperature sensitive compared to RM mitochondria.

Coupling ratios (ACR and RCR) were used to assess the degree of mitochondrial coupling at the different assay temperatures. In heart fibres there was both a significant decrease in ACR (4.75–1.93; p = 0.0093) and RCR (4.30–1.52; p = 0.0012), primarily driven by increases in State 2 and 4_o_ leak respiration rates as assay temperature increased from 20 to 30 °C (Table 1, Fig. 2C; Supplementary Fig. S1). The coupling ratios indicate that heart mitochondria were unable to maintain coupling as temperature increased from 20 to 26 °C. By comparison, there was a significant effect of assay temperature on RM RCR (2.57–1.62; p = 0.027), but not on RM ACR (3.96–2.51; p = 0.057). Though there were similar increases in State 4_o_ leak respiration in RM at a higher assay temperature (Supplementary Fig. S1), a posthoc Dunn’s multiple comparison test did not reveal significant differences in RM RCR at the three assay temperatures. Thus, the effect of temperature on coupling appears to be more subtle in RM than in the heart, which again supports heart mitochondria being more temperature sensitive compared to RM mitochondria.

**Table 1:** Respiratory flux ratios of permeabilized heart and RM fibres assayed at 20, 26, and 30 °C (Kruskal–Wallis test with Dunn’s multiple comparisons).

Respiratory flux ratios	Heart			Red muscle		
	20 °C	26 °C	30 °C	20 °C	26 °C	30 °C
**ACR**						
Complex I-stimulated State 3/State 2	4.75 ± 1.01^a^	3.22 ± 0.21^ab^	1.93 ± 0.24^b^	3.96 ± 0.76^e^	5.30 ± 1.70^e^	2.51 ± 0.40^e^
**RCR**						
Complex I & II-stimulated State 3/State 4_o_	4.30 ± 1.02^c^	1.95 ± 0.12^ cd^	1.52 ± 0.14^d^	2.57 ± 0.39^f^	2.42 ± 0.27^f^	1.62 ± 0.29^f^

Sample size for heart = 4, 3, 5 and for RM = 4, 3, 3 for data at 20, 26, and 30 °C, respectively.

Uncoupling increases as temperature rises because membrane integrity is compromised, and a larger portion of respiration rate is used to compensate for proton leak^16–18^. Eventually uncoupling negatively affects phosphorylation efficiency and induces cytochrome *c* release. This initiates the cell death cascade. In heat-stressed fish, it has been shown that mitochondria lose integrity at temperatures below the temperature at which the animal experiences heart failure^14^. However, increased uncoupling can also have beneficial effects. A high proton-motive force can stimulate reactive oxygen species (ROS) production from mitochondrial sites^19^. Excess ROS not scavenged by antioxidant mechanisms can then accumulate and lead to tissue oxidative damage. Increased uncoupling can help to dissipate the proton-motive force and lower the drive for ROS production^19^.

Similar to other species, mahi mitochondria show greater dependence on complex I rather than complex II in both heart and RM^16,17^. Complex I flux in both tissues makes up the majority of state 3 respiration rate when expressed to ETS capacity (73 and 79% respectively for heart and RM; Fig. 2A,D). The subsequent addition of succinate to stimulate combined complex I and II flux afterwards only increases respiration rate by a small portion (by 9 and 13%; Fig. 2B,E), indicating that the contribution of complex II is relatively low. A greater dependency on complex I flux results in a higher phosphorylation efficiency as complex I is a proton pump while complex II is not, and so complex II does not contribute to the proton gradient that stimulates ATP synthesis. When combined, complex I and II substrates gives a more physiologically relevant scenario of mitochondrial flux. Whereas in the heart, combined complex I and II flux shows temperature dependent increases (increasing from 82% at 20 °C to 92% at 26 °C). RM at 20 °C state 3 respiration rate already matches ETS capacity which suggests that RM did not have much reserve ETS capacity (Fig. 2E). These results indicate that RM likely relies on O_2_-independent ATP generation pathways (e.g. glycolysis) at a lower temperature than heart.

Temperature sensitivity of mitochondrial respiration can heavily depend on the flux pathways^16,20^. Although this was not explicitly investigated in this present study, this could be due to tissue specific differences in not only the ETS complexes themselves. Previous studies have shown tissue and species specificity in temperature sensitivity of individual mitochondrial complexes assayed in vitro^21,22^. Additionally, enzymes and pathways upstream of the ETS can also influence temperature sensitivity of ETS flux pathway. Rat heart mitochondria respiration showed high temperature sensitivity of pyruvate-driven respiration (for complex I) and was affected by the activity of pyruvate dehydrogenase at cooler temperatures^23^. How these factors could influence temperature sensitivity of mitochondrial function in an active pelagic fish will be interesting to investigate in detail.

Many species of pelagic fishes routinely forage at depths of > 300 m, and thus experience a wide temperature range in the wild (2.8–30 °C)^6–11^. A previous study comparing cardiac function between several pelagic fish species (bigeye tuna, yellowfin tuna, and mahi) showed that the temperature sensitivity was species specific, despite their similar lifestyles^8^. In mahi, heart contractile force decreases as temperatures increase from 22 to 25 °C suggesting that heart performance decreases above 22 °C. This is consistent with observations from the present study showing that heart mitochondria are significantly uncoupled at 26 °C compared to those assayed at 20 °C^8^. If mahi are indeed able to traverse through waters of 30 °C, our results show that even at 26 °C there is a substantial loss in phosphorylation efficiency in heart mitochondria.

### Uncoupling in heart fibres not reversible with acute 30 °C temperature challenge

We next investigated whether this uncoupling effect at high temperatures was reversible in both heart and RM mitochondria by examining their responses to in vitro acute temperature challenges. Based on previous studies of tagged fish, this acute temperature challenge is likely to be ecologically relevant (e.g. on v-type dives), but also serves to test the thermal limits of muscle fibres in vitro. For the acute temperature challenge, the state 3 and state 4_o_ respiration rates were compared between two treatments where (1) fibres experienced a 30–20 °C transition, and (2) where fibres were subjected to a 20–30 °C transition in vitro. The rate of temperature change selected, 1 °C/min, mimicked that of observed vertical dives made by a tagged mahi^6,7,10^. If mitochondria were able to acutely respond to temperature changes, and the effects on mitochondria were solely due to thermodynamic effects on reaction rates, then it would be predicted that fibres ending the challenge at a lower temperature (30–20 °C) would have lower respiration rates than fibres ending the challenge at a higher temperature (20–30 °C). This did not seem to be the case as there were no significant differences in either state 3 or state 4_o_ respiratory states between the two acute temperature treatments in either tissue. However, the general trends were similar, where the 30–20 °C treatment resulted in a higher respiration rate than the 20–30 °C treatment. As well, the coupling ratios of mitochondria after acute temperature change were low (RCR values between 1.20 and 1.83; data not shown), indicating a decrease in coupling. It is possible that the increase in uncoupling is due to a disruption of membrane integrity leading to membrane leak with high temperatures^18^. These results suggest that mitochondria were unable to maintain function in an in vitro acute temperature challenge.

### Summary

The findings from this study suggest that mahi heart tissue were more temperature sensitive than RM. Though the differences were observed at the fibre level, it would be important to determine how the differences in mitochondria function manifest at the organ and whole animal level under thermal stress. Moreover, it will be important in future studies to understand how acute temperature changes on heart mitochondrial function could subsequently affect susceptibility to other environmental stressors becoming more prominent in the mahi habitat, such as pollutants (e.g. polycyclic aromatic hydrocarbons^24^) and reduced oxygen availability.

## Methods

### Animal care

Young adult hatchery raised mahi (*F1* generation) were housed in two 3,000 L cylindrical fiberglass tanks supplied with filtered flow-through seawater (26 ± 1 °C) at the University of Miami Experimental Hatchery. Fish were maintained under a natural photoperiod and fed daily with a mixture of squid, mackerel, sardines, and pelletized diets, but fasted for 24 h before experiments.

### Fibre permeabilization

On each day of experiments, fish (0.33 ± 0.02 kg) were removed from their tanks, stunned by concussion, and euthanized by spinal severance. Heart and RM fibres were quickly excised and transferred to separate tubes containing ice-cold BIOPS solution (2.77 mM CaK_2_EGTA, 7.23 mM K_2_EGTA, 5.77 mM Na_2_ATP, 6.56 mM MgCl·6H_2_O, 20 mM taurine, 15 mM Na phosphocreatine, 20 mM imidazole, 0.5 mM dithiothreitol, 50 mM MES hydrate, 1 g/L bovine serum albumin, at pH 7.1). The procedure for permeabilizing heart and RM fibres is described in Chung et al.^3^. After permeabilization, fibres were kept in ice-cold MiR05 (0.5 mM EGTA, 3 mM MgCl_2_.6H_2_O, 60 mM K-lactobionic acid, 20 mM taurine, 10 mM KH_2_PO_4_, 20 mM HEPES, 110 mM sucrose, 1 g/L BSA, at pH 7.1^25^). Tissues were blotted dry and weighed before respiratory flux measurements.

### Respiratory flux measurements

**Figure 1:**
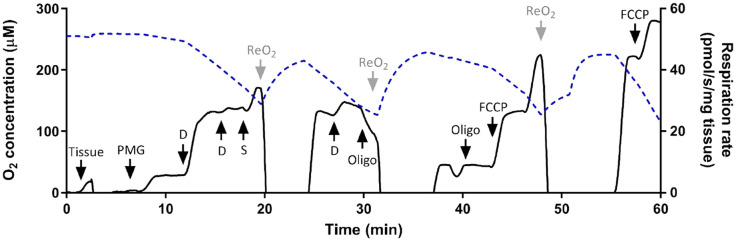
Representative trace for substrate utilization inhibitor titration (SUIT) protocol for permeabilized muscle fibres; the oxygen concentration trace (dotted blue line) is on the left y-axis and respiration rate trace (black line) on the right y-axis. *PMG* pyruvate, malate, glutamate, *D* ADP, *Succ* succinate, *Oligo* oligomycin, *ReO*_*2*_ reoxygenation; substrate or drug concentrations are detailed in methods.

High resolution respirometry was performed on permeabilized tissues from both heart and RM from each individual using the Oroboros oxygraph (Innsbruck, Austria) at three temperatures, 20, 26 and 30 °C. The polarographic oxygen sensors were calibrated daily with air saturated and anoxic MiR05 buffer at each of the three assay temperatures. To assess mitochondrial function, a substrate uncoupler inhibitor titration (SUIT) protocol was used (see representative trace in Fig. 1). To start, fibres were introduced into the 2 mL oxygraph chamber with MiR05 buffer stabilized at the assay temperature. First, substrates donating electrons to complex I were added (5 mM pyruvate, 1 mM malate, and 10 mM glutamate) to assess complex I-stimulated state 2 leak respiration, followed by 1 mM ADP to assess complex I-stimulated state 3 respiration. 10 mM succinate was then added to donate electrons to the electron transport system (ETS) via complex II to measure complex I and II-stimulated state 3 respiration. As ATPases are present in permeabilized fibres, 2–3 2.5 µM additions of oligomycin were used to induce state 4 leak respiration (from here on referred to as state 4_o_). Finally, 0.5 µM of carbonyl cyanide 4-(trifluoromethyoxy) phenylhydrazone (FCCP) was titrated stepwise to uncouple mitochondria and assess ETS capacity. 10 µM cytochrome *c* was used to check for membrane integrity (one fish was excluded from the analysis at 30 °C because membrane integrity appeared compromised, as evidenced by a greater than 10% increase in respiration rate with cytochrome *c* addition). The O_2_ concentration was never allowed to drop below 150 µM to prevent fibres from experiencing hypoxia; the suspension was re-oxygenated by introducing an air bubble into the chamber.

**Figure 2:**
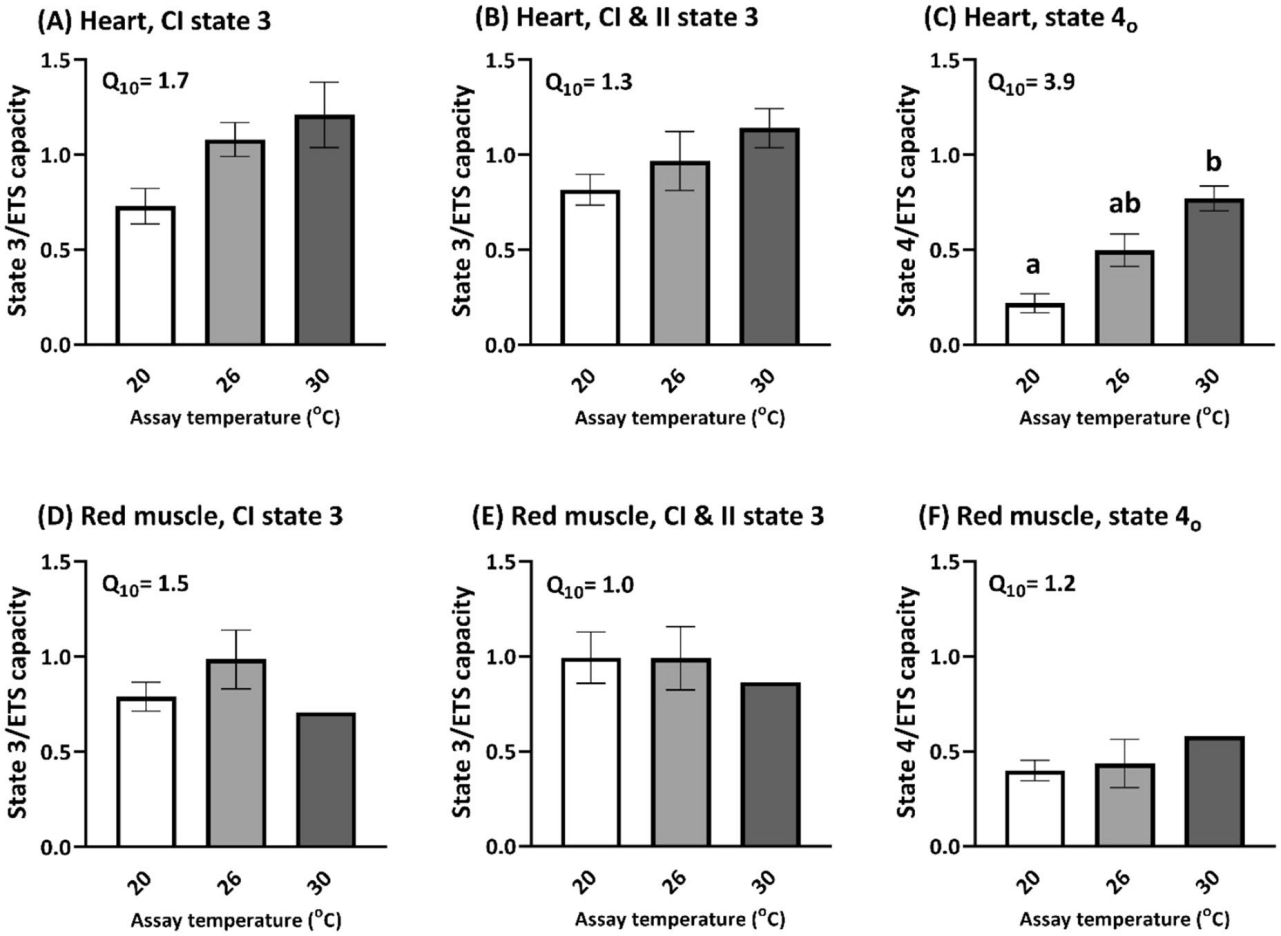
The effect of temperature (assayed at 20, 26, and 30 °C) on heart and RM permeabilized fibre respiration rates expressed to FCCP-uncoupled respiration rate representing ETS capacity; (**A**) heart complex I-stimulated state 3, (**B**) heart complex I and II-stimulated state 3, (**C**) heart oligomycin-induced state 4, (**D**) RM complex I-stimulated state 3, (**E**) RM complex I and II-stimulated state 3, and (**F**) RM oligomycin-induced state 4. The temperature coefficient (Q_10_) calculated using respiration rates at 20 and 26 °C are indicated in the panels. The Kruskal–Wallis test was used to test for statistical significance, and where p < 0.05 the Dunn’s multiple comparisons test was used. Different letters indicate significant differences. See Supplementary Fig. S1 for respiration rates expressed to tissue weight.

To assess the ability of mitochondria to tolerate acute temperature changes, we used a modified SUIT protocol (see representative trace in Fig. 2). After assessment of complex I-stimulated respiratory states 2 and 3, and complex I and II-stimulated respiratory state 3, the temperature of the chambers was changed stepwise at 1 °C/min (from 20 to 30 °C or from 30 to 20 °C). During this temperature change, the chamber was opened to allow for air mixing to prevent limiting O_2_ supply to the tissue. Once the temperature stabilized, the chamber was closed and the respirometer signal allowed to stabilize (for ~ 10 to 15 min). After signal stabilization, additional ADP was injected to ensure saturating levels, followed by measurement of state 4_o_ with oligomycin, membrane integrity with 10 µM cytochrome *c*, and ETS capacity with FCCP. To account for changes in O_2_ solubility with different temperatures, the measurements at each temperature were corrected to the respective temperature calibrations obtained that day.

### Data and statistical analysis

**Figure 3:**
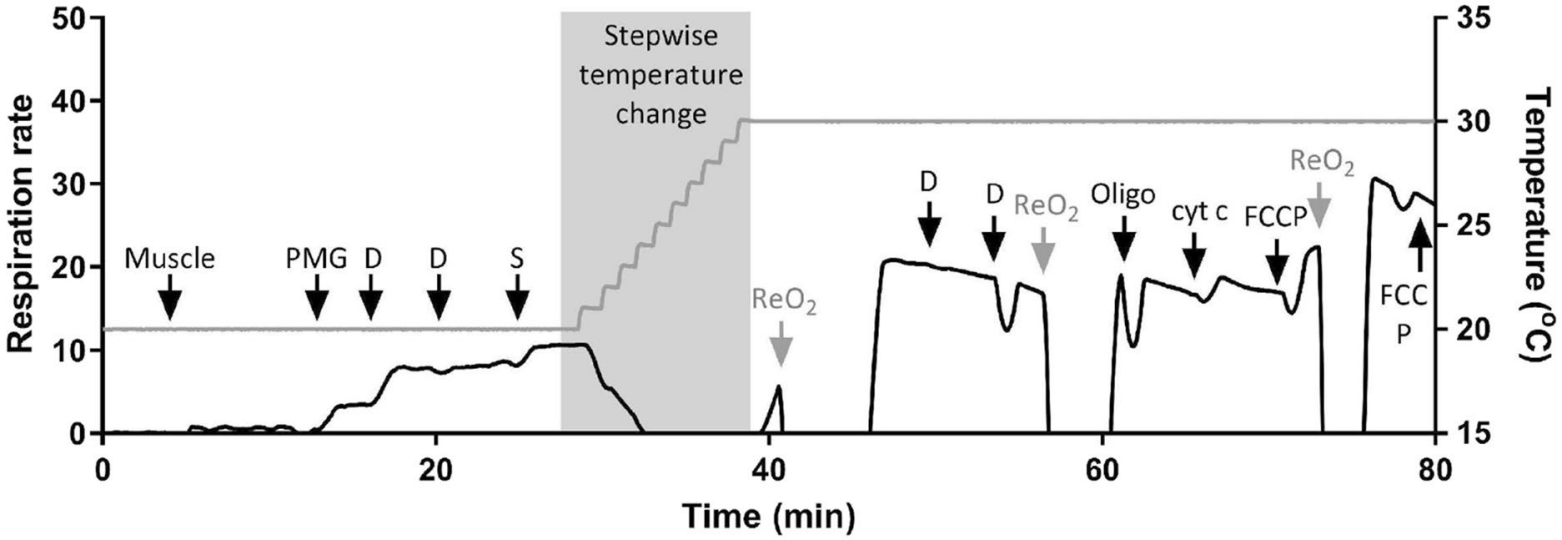
Representative trace for in-chamber acute temperature challenge; the respiration rate trace is on the left y-axis (black line) and temperature trace on the right y-axis (gray line). *PMG* pyruvate, malate, glutamate, *D* ADP, *Succ* succinate, *Oligo* oligomycin, *cyt c* cytochrome *c*, *ReO*_*2*_ reoxygenation; substrate or drug concentrations are detailed in methods.

**Figure 4:**
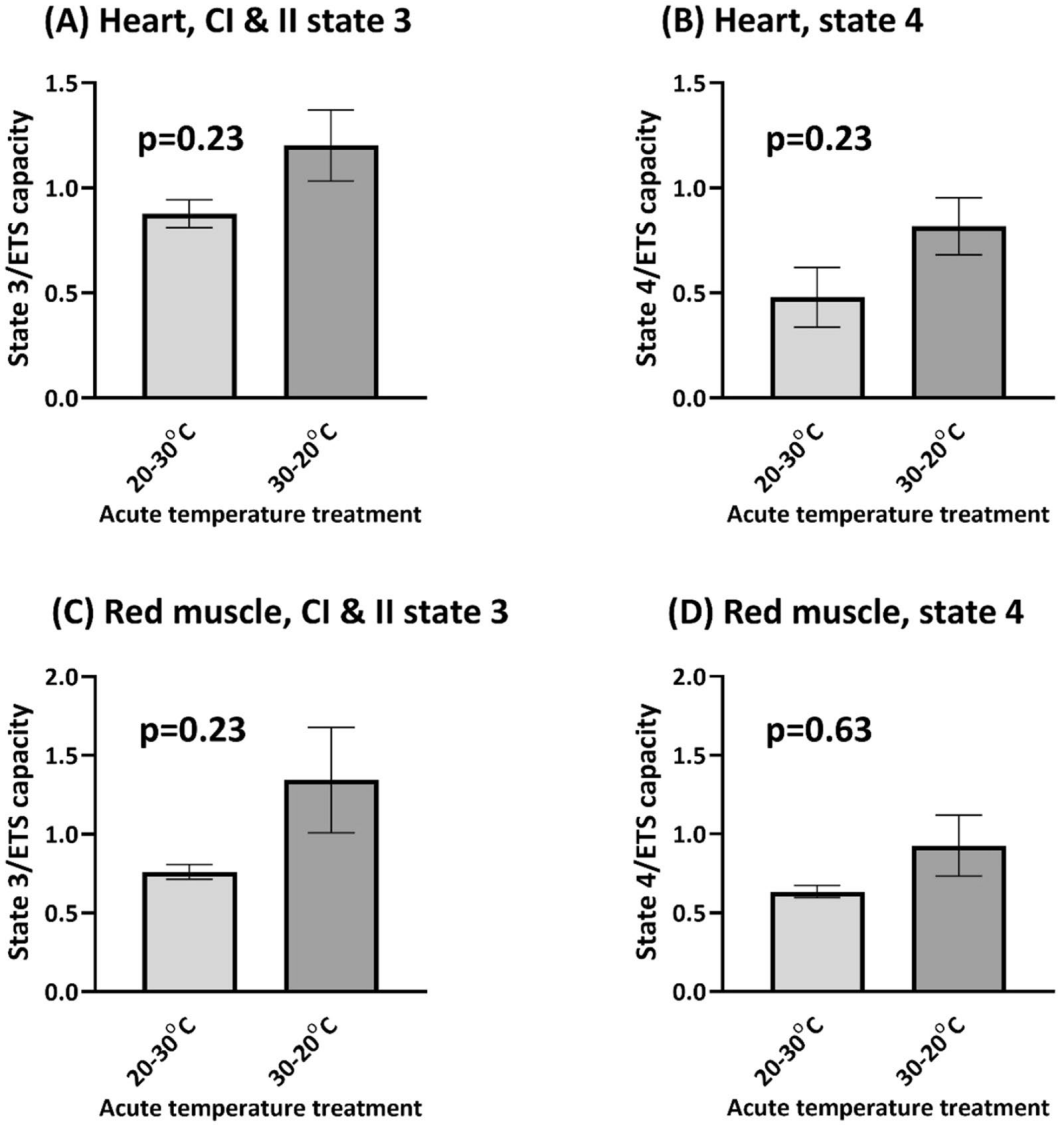
Effect of acute temperature challenge (from 20 to 30 °C, or 30 to 20 °C in vitro; see Fig. 3 for representative trace) on heart and RM permeabilized fibres respiration rates (expressed to ETS capacity); (**A**) heart complex I and II-stimulated state 3, (**B**) heart oligomycin-induced state 4, (**C**) RM complex I and II-stimulated state 3, and (**D**) RM oligomycin-induced state 4. Results from unpaired t-tests are indicated in the figure.

Mitochondrial respiration rates were expressed to ETS capacity (FCCP uncoupled respiratory state) to investigate changes in phosphorylation capacity (respiration rates expressed to tissue weight can be found in Supplementary Material). Statistical analysis was carried out using the Kruskal–Wallis tests, and significant effects were further tested with Dunn’s multiple comparisons. The temperature coefficient (Q_10_) values were calculated using respiration rates at 20 and 26 °C using the formula Q_10_ = ${(\frac{{R}_{2}}{{R}_{1}})}^{\left(\frac{10}{{T}_{2}-{T}_{1}}\right)}$, where R is the reaction rate and T represents the temperature of the reaction. The acceptor control ratio (ACR) was determined as the ratio of complex I-stimulated state 3 to complex I-stimulated state 2 respiration rate. The respiratory control ratio (RCR) was determined as the ratio of complex I and II-stimulated state 3 to state 4_o_ respiration rate. Complex I and II-stimulated state 3 respiration rate was expressed to ETS capacity (FCCP uncoupled respiratory state) to investigate phosphorylation capacity (Table 1). Differences between the three assay temperatures on ACR and RCR was tested using Kruskal–Willis tests with Dunn’s multiple comparisons. It should be noted that a sample of RM fibre assayed at 30 °C showed a large cytochrome *c* effect (where respiration rate increased > 10% and was subsequently removed from analysis), which indicates that there was outer membrane damage (Fig. 2C,D). Unpaired t-tests were used to analyze the effects of the in vitro acute temperature challenge on state 3 and 4_o_ (Figs. 3, 4).

### Ethics

All experimental procedures in this study were approved by the University of Miami’s Institutional Animal Care and Use committee (IACUC Protocol number 15-190), following all applicable laws and regulations.

## Supplementary information

Supplementary file1

## Data Availability

Data are publicly available through the Gulf of Mexico Research Initiative Information and Data Cooperative (GRIIDC) at https://data.gulfresearchintiative.org (Doi: https://doi.org/10.7266/n7-wyp8-rc91).
