# Supplementary material for: Temperature sensitivity differs between heart and red muscle mitochondria in mahi-mahi (Coryphaena hippurus)
Source: Sci Rep. 2020 Sep 10;10:14865. doi: 10.1038/s41598-020-71741-0 (PMC7484784; doi:10.1038/s41598-020-71741-0)
Supplement: Supplementary file 1 — Supplementary file1 [file 41598_2020_71741_MOESM1_ESM.docx]

Temperature sensitivity differs between heart and red muscle mitochondria in mahi-mahi (*Coryphaena hippurus*).

Gigi Y. Lau*^1^, Georgina K. Cox*^2^, John D. Stieglitz^3^, Daniel D. Benetti^3^, Martin Grosell^2^

* denotes equal contribution

Corresponding author: G.Y. Lau (glau@zoology.ubc.ca) and G.K. Cox (georgina@georginacox.ca)

Affiliations:

1.     Department of Zoology, University of British Columbia, 6270 University Blvd, Vancouver, BC, Canada, V6T 1Z4

2.     Department of Marine Biology and Ecology, Rosenstiel School of Marine and Atmospheric Science, University of Miami, 4600 Rickenbacker Causeway, Miami, FL, United States, 33149

3.     Department of Marine Ecosystems and Society, Rosenstiel School of Marine and Atmospheric Science, University of Miami, 4600 Rickenbacker Causeway, Miami, FL, United States, 33149


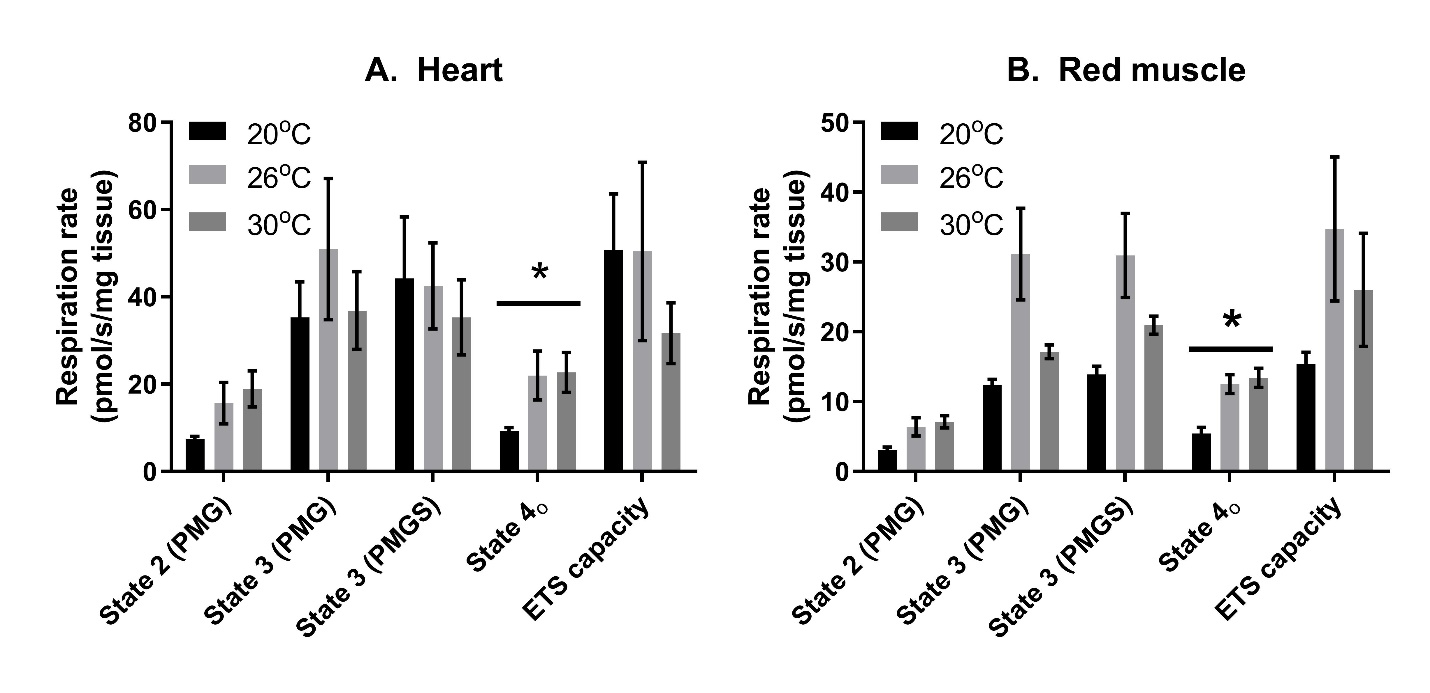


**Supplementary Figure 1.** Heart and RM respiration rates from SUIT protocol (representative trace in Supplementary Fig.1) at three different assay temperatures: 20, 26, 30^o^C.
